# Structural Insights into M1 Muscarinic Acetylcholine Receptor Signaling Bias between Gαq and β-Arrestin through BRET Assays and Molecular Docking

**DOI:** 10.3390/ijms24087356

**Published:** 2023-04-16

**Authors:** Dongxue Wang, Yunjin Yao, Shiqi Wang, Yifei Hou, Lanxue Zhao, Hao Wang, Hongzhuan Chen, Jianrong Xu

**Affiliations:** 1 Institute of Interdisciplinary Integrative Medicine Research, Shanghai University of Traditional Chinese Medicine, Shanghai 201203, China; wangdongxue919912@126.com (D.W.);; 2 Academy of Integrative Medicine, Shanghai University of Traditional Chinese Medicine, Shanghai 201203, China; 3 Department of Pharmacology and Chemical Biology, Shanghai Jiao Tong University School of Medicine, Shanghai 200025, China; 4 Shanghai Universities Collaborative Innovation Center for Translational Medicine, Shanghai Jiao Tong University School of Medicine, Shanghai 200025, China; 5 Shanghai Frontiers Science Center of TCM Chemical Biology, Shanghai University of Traditional Chinese Medicine, Shanghai 201203, China

**Keywords:** M1 muscarinic acetylcholine receptor, signaling bias, Gαq, β-arrestin, BRET, molecular docking

## Abstract

The selectivity of drugs for G protein-coupled receptor (GPCR) signaling pathways is crucial for their therapeutic efficacy. Different agonists can cause receptors to recruit effector proteins at varying levels, thus inducing different signaling responses, called signaling bias. Although several GPCR-biased drugs are currently being developed, only a limited number of biased ligands have been identified regarding their signaling bias for the M1 muscarinic acetylcholine receptor (M1mAChR), and the mechanism is not yet well understood. In this study, we utilized bioluminescence resonance energy transfer (BRET) assays to compare the efficacy of six agonists in inducing Gαq and β-arrestin2 binding to M1mAChR. Our findings reveal notable variations in agonist efficacy in the recruitment of Gαq and β-arrestin2. Pilocarpine preferentially promoted the recruitment of β-arrestin2 (∆∆RAi = −0.5), while McN-A-343 (∆∆RAi = 1.5), Xanomeline (∆∆RAi = 0.6), and Iperoxo (∆∆RAi = 0.3) exhibited a preference for the recruitment of Gαq. We also used commercial methods to verify the agonists and obtained consistent results. Molecular docking revealed that certain residues (e.g., Y404, located in TM7 of M1mAChR) could play crucial roles in Gαq signaling bias by interacting with McN-A-343, Xanomeline, and Iperoxo, whereas other residues (e.g., W378 and Y381, located in TM6) contributed to β-arrestin recruitment by interacting with Pilocarpine. The preference of activated M1mAChR for different effectors may be due to significant conformational changes induced by biased agonists. By characterizing bias towards Gαq and β-arrestin2 recruitment, our study provides insights into M1mAChR signaling bias.

## 1. Introduction

The G-protein-coupled receptors (GPCRs) play crucial roles in facilitating cellular communication, which regulates various important physiological processes. In humans, over 800 GPCRs are responsible for detecting an extensive range of extracellular stimuli, such as hormones, ions, light, and neurotransmitters. By triggering a variety of intracellular signaling pathways, GPCRs prompt cellular responses [1]. It is worth noting that GPCRs are not only the most significant group of membrane targets for drugs approved by the US Food and Drug Administration (FDA), but are also one of the most under-researched drug targets in the human genome [2,3].

Initially, it was believed that G-protein-mediated signaling was the only pathway utilized by GPCRs. However, the discovery of β-arrestin-mediated signaling revealed an alternative mechanism that operates independently of G proteins and contributes to the termination of the signaling cascade [4,5]. When an agonist binds to the GPCR, the receptor undergoes conformational changes that are recognized by the G protein. Upon activation, the G protein dissociates from the receptor and recruits β-arrestin by phosphorylating the receptor via GPCR kinase. Consequently, the G protein is unable to bind to the receptor, resulting in signal termination [6]. In recent years, study findings have revealed that GPCR signaling is a complex process that produces multiple downstream effects of varying potency. When ligands targeting GPCR preferentially activate specific downstream signaling pathways while activating other signaling pathways less efficiently, this signal transduction phenomenon is known as the “signaling bias” [7]. The discovery of signaling bias has shifted the focus of GPCR research toward the development of biased ligands that bind receptors to specific effectors, thus enhancing particular signaling pathways [6].

The muscarinic acetylcholine receptors (mAChRs) are a group of GPCRs that are activated by the neurotransmitter acetylcholine. Among these mAChRs, the M1 subtype (M1mAChR) is considered to be an important therapeutic target for Alzheimer’s disease (AD) and other neurodegenerative diseases [8]. The primary function of M1mAChR is signal transduction through downstream coupling with Gαq, along with β-arrestin signaling. The activation of the Gαq pathway has the potential to improve cognitive deficits and reduce the formation of Aβ (Amyloid β-protein, Aβ) plaques [9]. However, it can also lead to unwanted side effects, such as salivation, seizures, and hyperactivity [10]. On the other hand, the recruitment of β-arrestin-dependent signaling has been shown to enhance learning and memory, and modulate anxiety-related behaviors, minimizing M1mAChR-mediated adverse reactions [11]. Currently, several GPCR-biased ligands are being developed, including G-protein-biased and β-arrestin-biased ligands, with some already in clinical use [12]. However, only a limited number of ligands have been identified for their signaling bias towards M1mAChR, and the underlying mechanism of M1mAChR signaling bias is not yet well understood [13].

Though the calcium immobilization assay has been commonly used to assess the coupling of Gαq to the receptor, it does not measure the direct binding to the receptor and may be affected by the complex regulatory mechanisms of calcium influx, such as Gαi activation [14]. Recently, novel techniques, such as surface plasmon resonance and flow cytometry, have been developed to measure real-time interactions between GPCRs and G proteins [15,16]. However, these methods are performed outside the cell and cannot determine the effects of other factors inside the cell in real time. To address this limitation, using the bioluminescence resonance energy transfer (BRET) approach, biosensors have been developed to more directly, sensitively, and accurately measure GPCR activity in living cells [17]. BRET is based on the Förster resonance energy transfer that occurs in some marine species (e.g., Renilla reniformis), which results in a nonradiative energy transfer [18]. The energy donor is a luciferase that emits light in the presence of the corresponding substrate. The energy acceptors are fluorescent proteins that absorb a certain wavelength of light and reemit it at a longer wavelength. Energy transfer requires the donor’s emission spectrum to overlap with the acceptor’s excitation spectrum [19]. Thus, we can fuse the target proteins with a luciferase as a donor and a fluorescent acceptor to construct cDNAs, and then transfect these constructs into cells for expression. If the two fusion proteins do not interact, only the light emitted by the energy donor during substrate conversion can be detected. If the two fusion proteins interact and the distance between the energy donor and acceptor is less than 10 nm, resonant energy transfer occurs, and an additional light signal corresponding to the re-emission of the acceptor can be detected [20].

**Figure 1:**
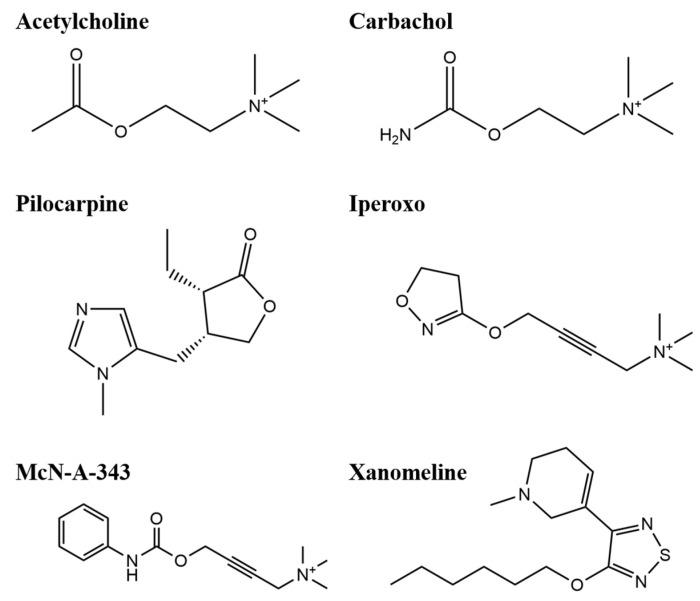
The 2D chemical structures of the six agonists.

In this study, we utilized BRET sensors to investigate the signaling bias of M1mAChR ligands for Gαq and β-arrestin, and employed molecular docking approaches to analyze the structural features underlying signaling biases. Six typical M1mAChR agonists, including acetylcholine (ACh), carbachol (CCh), Pilocarpine, Iperoxo, McN-A-343, and Xanomeline (Figure 1), were selected for this study. We aimed to enhance our understanding of the functionally selective drugs that target M1mAChR, thus facilitating the development of more specific and effective medicines.

## 2. Results

### 2.1. Efficacy of M1mAChR Agonists in Gαq-Binding Revealed by BRET Assays

**Figure 2:**
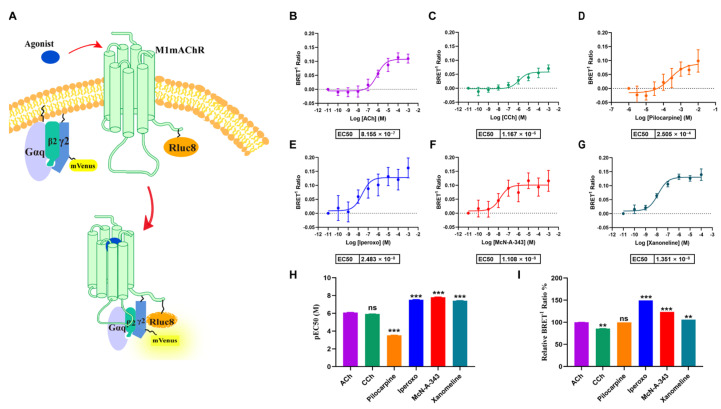
Efficacy of agonists in Gαq-binding to M1mAChR revealed by BRET assays. (**A**) Schematic illustration of bioluminescent resonance energy transfer (BRET) and dose-response curves for ACh- (**B**), CCh- (**C**), Pilocarpine- (**D**), Iperoxo- (**E**), McN-A-343- (**F**), and Xanomeline-induced (**G**) Gαq interactions with M1mAChR. (**H**) EC50 of agonists and (**I**) Emax of agonists (means ± S.E.M., *n* = 3). The statistical significance of the differences between the indicated groups and the ACh was assessed by one-way ANOVA, where *** *p* < 0.001, ** *p* < 0.01 is ns, not significant.

For many receptors including M1mAChR, it is well known that signal transduction upon G protein activation leads to a spare receptor phenomenon. That is to say, when agonists produce maximum effects, there could be some receptors unoccupied by the G protein and the occupation could vary for different agonists. Therefore, we evaluated the efficacy of different agonists in facilitating Gαq binding to M1mAChR using BRET-based measurements of human embryonic kidney 293T (HEK293T) cells. We observed a concentration-dependent increase in the ratio of mVenus fluorescence to Rluc bioluminescence following stimulation with a 10-fold gradient of ACh, which reflected the BRET response as a result of Gαq binding to M1mAChR (Figure 2A,B). We found that the other five agonists (Figure 2C–G) also produced a concentration-dependent rise.

Our results showed that Pilocarpine had the worst potency (EC50 = 250 μM), with an EC50 that was approximately 300 times higher than ACh (EC50 = 815 nM). On the contrary, McN-A-343, Iperoxo, and Xanomeline were more potent, with EC50 values of 11 nM, 24.8 nM, and 37 nM, respectively. These agonists also produced a higher maximum response, with increases of approximately 49% for Iperoxo, 23% for McN-A-343, and 6.8% for Xanomeline, compared to Ach. However, Pilocarpine required a concentration of 3 mM to reach the response level of Ach, while CCh showed a similar EC50 to Ach (Figure 2H,I). Our findings allowed us to compare the varied recruitment capacities of several ligands for Gαq following the activation of M1mAChR, with Pilocarpine having the lowest recruitment capacity, and McN-A-343 and Iperoxo having strong recruiting capabilities.

### 2.2. Kinetics of Gαq-M1mAChR Coupling Assessed by BRET Assays

**Figure 3:**
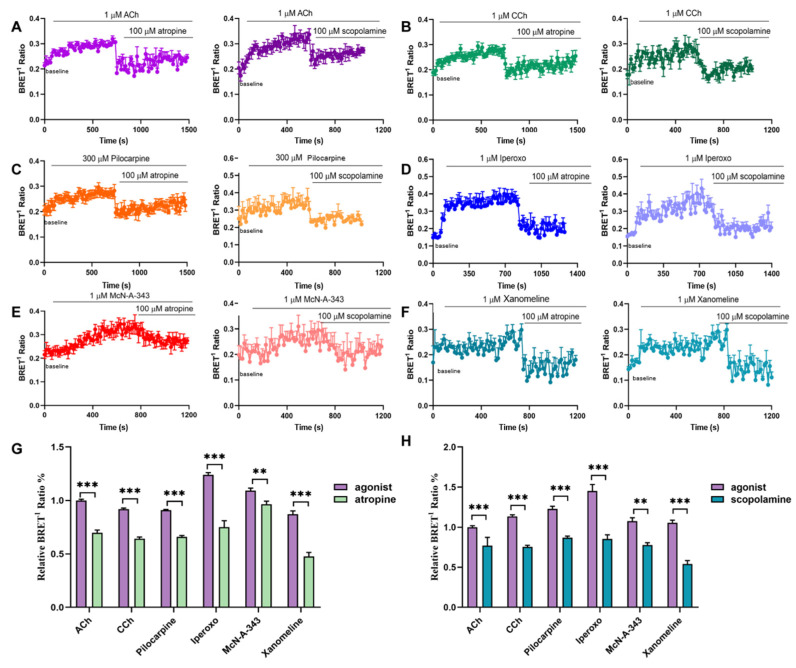
Analysis of antagonist effects of Gq-M1mAChR binding assessed by BRET assays. (**A**) ACh, (**B**) CCh, (**C**) Pilocarpine, (**D**) Iperoxo, (**E**) McN-A-343, (**F**) Xanomeline, (**G**) antagonistic potency of Atropine to agonists, and (**H**) antagonistic potency of Scopolamine to agonists. Data are means ± S.E.M. of 3–8 independent experiments performed in duplicate. The statistical significance of differences between the indicated groups was assessed by two-way ANOVA, where ** *p* < 0.01, *** *p* < 0.001.

In this study, we investigated the response kinetics of six agonists by measuring the changes in the BRET ratio of M1mAChR. The local perfusion of the agonist or antagonist resulted in either an increase or decrease in BRET, respectively (Figure 3A–F). To conduct a robust assessment of the agonist efficacy, we used 1 μM of agonist (ACh, CCh, Iperoxo, McN-A-343, and Xanomeline) and 300 μM of Pilocarpine, and kept them as constant for comparison in the Gαq recruitment assays. The response increased gradually after applying a specific quantity of agonist and reached its maximum at approximately 400 s. However, the addition of an antagonist (Atropine or Scopolamine) at 100 μM led to the rapid return of the BRET response to its baseline (Figure 3A–F). We analyzed the mean response for 100 s before or after the injection of the antagonist. Atropine showed the least potent antagonistic effect on McN-A-343, with a 12% decrease, while it had the strongest antagonistic effect on Xanomeline, with a 46% reduction (Figure 3G). For the other agonists, there was a decrease of approximately 30% (Figure 3G). Scopolamine also showed the strongest antagonistic effect on Xanomeline, with a reduction of approximately 49%, followed by Iperoxo, with a reduction of approximately 41% (Figure 3H). The antagonistic potency of the other four agonists was around 20–30% (Figure 3H). Our results demonstrate that the use of antagonists provides further insight into the different potencies and recruitment abilities of various agonists.

### 2.3. Efficacy of Agonists in β-Arrestin2-Binding to M1mAChR Revealed by BRET Assays

**Figure 4:**
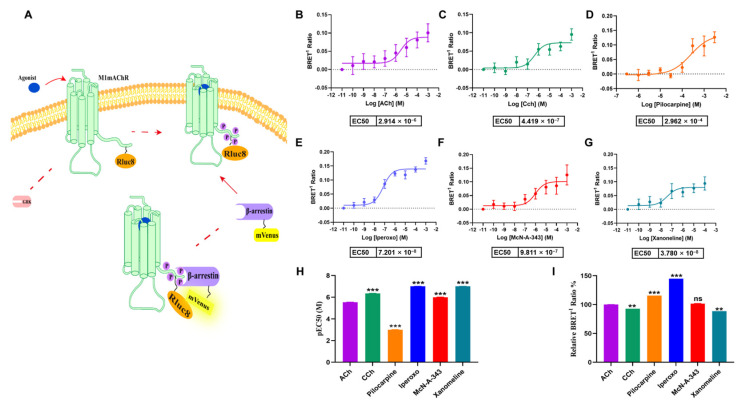
Efficacy of agonists in β-arrestin2 binding to M1mAChR revealed by BRET assays. (**A**) Schematic illustration of bioluminescent resonance energy transfer (BRET) and dose-response curves for ACh- (**B**), CCh- (**C**), Pilocarpine- (**D**), Iperoxo- (**E**), McN-A-343- (**F**), and Xanomeline-induced (**G**) β-arrestin2 interactions with M1mAChR. (**H**) EC50 of agonists and (**I**) Emax of agonists (means ± S.E.M., *n* = 3). The statistical significance of differences between the indicated group and the ACh was assessed by one-way ANOVA, where ** *p* < 0.01, *** *p* < 0.001 is ns, not significant.

Using the BRET assay, we measured the recruitment of β-arrestin by M1mAChR, as shown in Figure 4A. Our team previously developed a BRET system, with the fluorescent acceptor fused to the β-arrestin2 subtype. Similar to Gαq, the concentration-dependent increase in the ratio of mVenus fluorescence to Rluc bioluminescence, following the stimulation of cells with 10-fold gradients of the six agonists, indicated β-arrestin binding to M1mAChR (Figure 4B–G). Pilocarpine showed the lowest potency, with an EC50 of 296 μM, which is approximately 100 times that of ACh. The EC50 values of the other four agonists were lower than that of ACh, with Xanomeline at 13.5 nM, Iperoxo at 72 nM, CCh at 440 nM, and McN-A-343 at 980 nM. At the same time, compared to ACh, an increase in the maximum response level was exhibited by Iperoxo (45% for ACh) and McN-A-343 (1.7% for ACh). Surprisingly, relative to ACh, the Pilocarpine response showed an increase of 15%. Our findings suggest that different agonists activate M1mAChR with varying success to recruit β-arrestin for different signaling pathways. This implies that the recruitment of Gαq and β-arrestin by these agonists may show signaling bias.

### 2.4. Kinetics of β-Arrestin2-M1mAChR Coupling Assessed by BRET Assays

**Figure 5:**
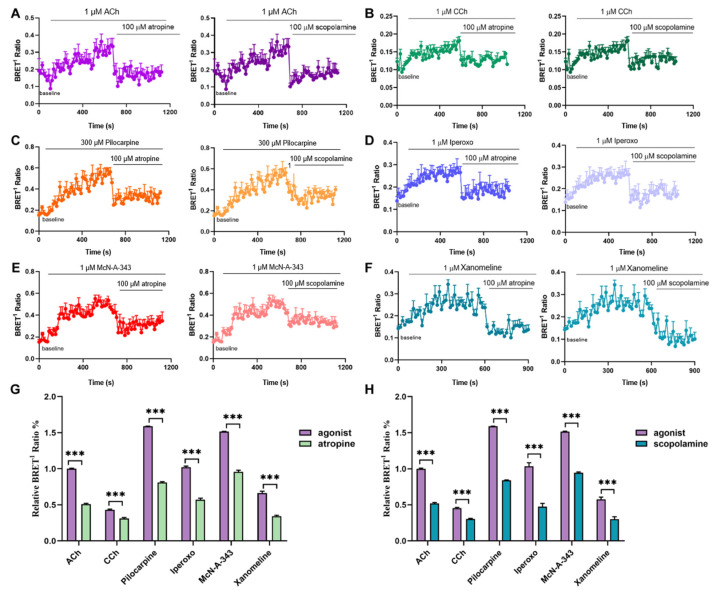
Analysis of antagonist effects on β-arrestin2-M1mAChR binding by BRET. (**A**) ACh, (**B**) CCh, (**C**) Pilocarpine, (**D**) Iperoxo, (**E**) McN-A-343, (**F**) Xanomeline, (**G**) antagonistic potency of Atropine to agonists, and (**H**) antagonistic potency of Scopolamine to agonists. Data are means ± S.E.M. of 3–8 independent experiments performed in duplicate. The statistical significance of differences between the indicated groups was assessed by two-way ANOVA, where *** *p* < 0.001.

The kinetic response of the agonists to β-arrestin recruitment was analyzed. The study revealed that the rapid local perfusion of agonists (ACh, CCh, Pilocarpine, Iperoxo, McN-A-343, and Xanomeline) or antagonists (Atropine and Scopolamine) resulted in increases or decreases in the BRET ratio of M1mAChR, respectively (Figure 5A–F). In the β-arrestin recruitment assays, 1 μM of agonist (ACh, CCh, Iperoxo, McN-A-343, and Xanomeline) or 300 μM of Pilocarpine was used and kept as constant, as in the case of Gαq, for comparison. The response gradually increased after applying a specific quantity of agonist, but when 100 μM of an antagonist (Atropine or Scopolamine) was added, the BRET response rapidly returned to its baseline. The response also increased gradually after the application of a specific amount of agonist, and most agonists reached a maximum after approximately 500 s, compared to Gαq. However, Xanomeline reached a maximum at approximately 300 s, followed by a gradual decrease in response (Figure 5F).

The mean response over 100 s before and after the injection of the antagonist was also analyzed. All six agonists were effectively antagonized by Atropine, although the antagonistic action on Iperoxo was much stronger (60%). The responses of Xanomeline and Pilocarpine were decreased by approximately 50% and 38% for McN-A-343, and 28% for CCh (Figure 5G). Scopolamine showed the strongest antagonistic effect on Iperoxo at 54%, followed by Xanomeline at 50%, Pilocarpine at 47%, ACh at 42%, McN-A-343 at 37%, and CCh at 32% (Figure 5H). The results showed that both Atropine and Scopolamine had relatively weak antagonistic effects on McN-A-343 and CCh. There was also a slight antagonistic effect on McN-A-343 in the detection of the Gαq pathway, indicating differences between McN-A-343 and the other agonists.

### 2.5. Determination of Gαq and β-Arrestin2 Pathway Activation by Commercial Methods

**Figure 6:**
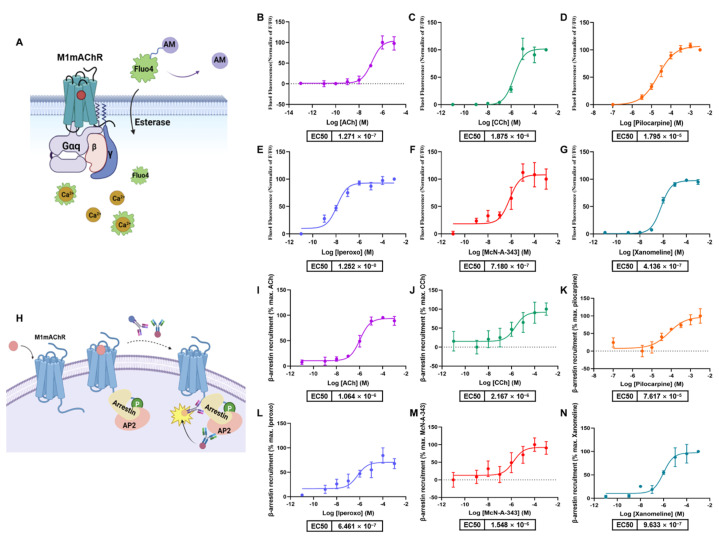
Determination of Gαq and β-arrestin2 recruitment to M1mAChR using commercial methods. (**A**) Schematic illustration of Fluo4-AM and dose-response curves for (**B**) ACh, (**C**) CCh, (**D**) Pilocarpine, (**E**) Iperoxo, (**F**) McN-A-343, and (**G**) Xanomeline, verified by Fluo4-AM. (**H**) Schematic illustration of FRET and dose-response curves for (**I**) Ach, (**J**) CCh, (**K**) Pilocarpine, (**L**) Iperoxo, (**M**) McN-A-343, and (**N**) Xanomeline, verified by FRET. Data are means ± S.E.M. of 3 independent experiments performed in duplicate.

The M1mAChR receptor primarily couples with Gαq, which connects it to several signaling pathways, such as PLC (phospholipase C, PLC), IP3 (inositol triphosphate, IP3), DAG (diacylglycerol, DAG), PKC (protein kinase C, PKC), and calcium signaling [21]. Additionally, the Gαq-PLC signaling pathway plays a role in the activation of cAMP (Adenosine 3’,5’-phosphate monohydrate, cAMP) via IP3-calcium release and calmodulin [9]. To evaluate the impacts of agonists on calcium immobilization, Fluo4 assays were conducted based on the principles shown in Figure 6A. The rank order of agonist affinity was determined using Fluo-4-AM (Figure 6B–G), and it was found to have a similar affinity ranking to that previously established using the BRET method.

To further analyze the signaling bias of the agonists, a β-arrestin recruitment kit was utilized based on HTRF (Homogeneous Time Resolved Fluorescence, HTRF) technology. The interaction between native β-arrestin2 and AP2 was detected using a Europium cryptate-labeled AP2 (Adaptin2, AP2) antibody (Europium donor) and a d2-labeled β-arrestin2 antibody (acceptor) (Figure 6H) [22]. The operational model of antagonism toward the β-arrestin2 pathway was fitted to the concentration–response curves in order to determine the EC50, which saw the following ranking in terms of potency: Iperoxo (EC50 = 12.5 nM) > Xanomeline (EC50 = 413 nM) > McN-A-343 (EC50 = 718 nM) > CCh (EC50 = 1.1 μM) > Pilocarpine (EC50 = 17.9 μM) (Figure 6I–N).

### 2.6. Evaluation of the Signaling Bias of M1mAChR Agonists

**Figure 7:**
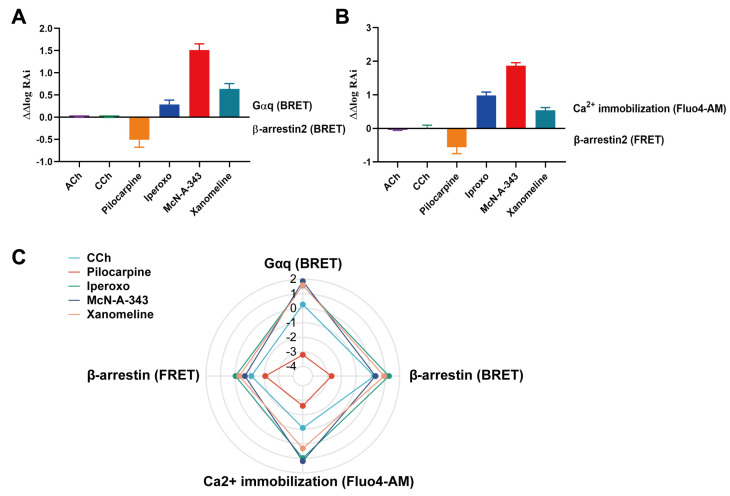
Evaluating the signaling bias of M1mAChR agonists. (**A**) Bias plots for ligands of M1mAChR using BRET assays. (**B**) Bias plots for ligands of M1mAChR obtained by commercial methods. (**C**) Comparing BRET assays and commercial methods in order identifying which agonist signal is preferred (relative intrinsic activity of each method). Data are means ± S.E.M. of 3–8 independent experiments performed in duplicate.

The potencies and efficacies of Gαq and β-arrestin2 were verified using BRET assays, as well as commercial methods, to monitor G protein binding and β-arrestin recruitment (refer to Figure 7). For M1mAChR, using the relative intrinsic activity (RAi) statistical bias factor, Pilocarpine showed a preference for the most effective β-arrestin2 recruitment (Figure 7A), with a bias factor of 0.32. In contrast, McN-A-343 (bias factor = 34), Xanomeline (bias factor = 4.5), and Iperoxo (bias factor = 2) exhibited very high preferential activity towards the recruitment of Gαq (Figure 7A), with no signal preference effect in the case of ACh or CCh.

The results based on the calcium assay and commercial β-arrestin recruitment method confirmed that Pilocarpine displayed a bias towards the recruitment of β-arrestin (Figure 7B), while McN-A-343, Xanomeline, and Iperoxo were predominantly biased towards Gαq (Figure 7B). Through a statistical analysis of the relative intrinsic activity, we found that the BRET assay could effectively highlight signaling bias differences when compared to the commercial method, and similar results were obtained (Figure 7C and Table 1).

### 2.7. The Interaction Underlying the Signaling Bias Observed in Molecular Docking

To investigate the mechanisms underlying the signaling bias among the ligands and receptors, we conducted docking studies of McN-A-343 and Pilocarpine in the models of M1mAChR, using structures of the receptor in complex with tiotropium in its inactive state and with the Gα11 complex in its active state. Both agonists were placed in the orthosteric ligand binding site, where they bind competitively with other orthosteric ligands.

**Figure 8:**
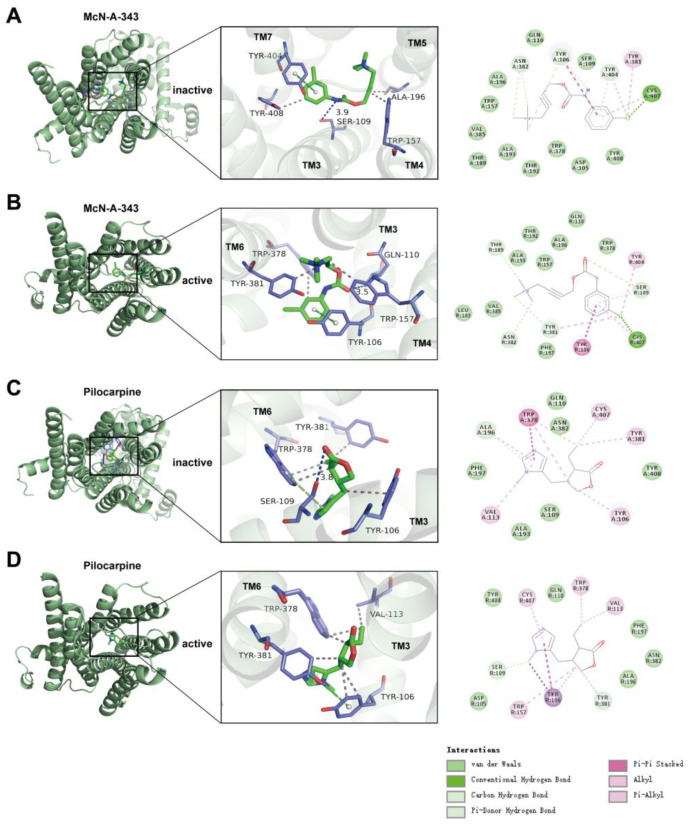
Interaction sites of agonists with M1mAChR. Binding site and interaction of McN-A-343 with inactive M1mAChR (**A**), Binding site and interaction of McN-A-343 with active M1mAChR (**B**), Binding site and interaction of Pilocarpine with inactive M1mAChR (**C**), and binding site and interaction of Pilocarpine with inactive M1mAChR (**D**). Dotted lines indicate the interaction between the ligand and the corresponding residues; while the line colours indicate the interaction types.

McN-A-343 was found to effectively bind to the active pocket of M1mAChR, with a binding energy of −7.5 kcal/mol. The compound was able to form hydrophobic interactions, π–π parallel interactions, and hydrogen bonds with protein binding sites. Specifically, it formed a hydrogen bond interaction with S109 of M1mAChR, with a distance of 3.9 Å. The hydrophobic functional group in McN-A-343 interacted with M1mAChR Y404, Y408, A196, and W157, forming hydrophobic interactions (Figure 8A). In both active and inactive states (Figure 8A,B), McN-A-343 formed a π–π conjugation with Y404 on M1mAChR to produce π–π contacts with Y106. These amino acids are located on TM3, TM4, TM5, and TM7, leading to certain conformational alterations in the transmembrane helix. It was previously reported that the active-state structure of M1AChR in complex with Gα11 showed a 5.7 Å inward movement at the extracellular end of TM6, relative to the inactive-state M1AChR, and accompanied by an outward movement of the cytoplasmic side of TM6 [23]. The binding of McN-A-343 to M1mAChR may cause conformational changes in TM and thus result in Gαq bias.

On the other hand, Pilocarpine bound to S109 of M1mAChR with a hydrogen bond, which had a distance of 3.8 Å and a binding energy of −7.0 kcal/mol. The binding pocket was created via hydrophobic interactions with Y381, W378, and Y106 of M1mAChR (Figure 8C). The primary hydrophobic interaction between M1mAChR and Pilocarpine, which was formed by Y381 and W378, and the π–π contact between the imidazole ring of Pilocarpine and W378, were both revealed by molecular docking. In both the active and inactive states of M1mAChR, Y106 has an important hydrophobic interaction with W378, which is the key to the binding of Pilocarpine; this is mainly located at TM3 and TM6 of M1mAChR (Figure 8C,D). It has been shown that proximity to TM3 and the displacement of TM6 favor the binding of the receptor to β-arrestin.

The key-acting amino acid of the agonist that shows bias towards Gαq is Y404, while the core-acting amino acids of Pilocarpine are W378 and Y381. Different core-acting amino acids may cause different conformational changes in TM, leading to different signaling biases.

## 3. Discussion

M1mAChR is predominantly expressed in the hippocampus and cortex, which are the primary centers for cognitive function and neuropsychological regulation, and are vital for learning and memory. Consequently, M1mAChR is implicated in various neurological disorders, and reduced M1mAChR signaling has been closely associated with cognitive decline in AD [24]. The development of drugs targeting M1mAChR has been a focus of therapy for neurological disorders. However, given the potential for the harmful overactivation of the receptor by orthosteric or allosteric ligands, drug candidates must exhibit a signaling bias to avoid off-target adverse effects. In the field of GPCR pharmacology, it is widely accepted that different agonists can stabilize distinct conformational states of receptors, and several studies have suggested that these states may be responsible, in part, for the resulting selective activation of signaling pathways [25]. However, a direct comparison of agonist biases concerning Gαq and β-arrestin recruitment to M1mAChR has not yet been conducted.

Rather than analyzing downstream signaling bias, we assessed the direct recruitment of Gαq and β-arrestin2 to M1mAChR using a BRET approach, in addition to several commercial assays. To investigate signaling bias, we fused the fluorescent acceptor mVenus with the Gγ2 subunit and β-arrestin2, and the donor Rluc8 with M1mAChR. Although our BRET assay yielded a higher relative intrinsic activity compared to commercial methods, its effect in generating signaling bias was consistent (Figure 7). The reason that we utilized BRET assays is that BRET can directly detect the coupling of M1mAChR to Gαq or β-arrestin, whereas Fluo4-AM can only detect intracellular Ca^2+^ mobilization indirectly by activating Gαq and by triggering Ca^2+^ release, which is caused by phospholipase C [9]. The FRET (fluorescence resonance energy transfer, FRET) assay detected the recruitment of β-arrestin by labeling the acceptor on β-arrestin and labeling the donor on AP2 downstream of β-arrestin [26]. However, the interaction of β-arrestin with AP2 occurs not only at the cell membrane, but also at the subcellular organelle membranes (such as endosomes) [27]. As a result, the commercial methods differ from the BRET assay not only in time, but also in space. Recent studies have shown that biosensors using BRET can more accurately, sensitively, and promptly assess G protein activity in living cells [17]. It has also been reported that BRET can be used to identify the spatial and temporal biases of GPCR signals [1]. Our results also indicate the advantages of BRET in studying GPCR signaling and bias.

Comparing the agonists in terms of potency, it is interesting to note that different agonists showed similar efficacy in Gαq and β-arrestin recruitment (Figure 2 and Figure 4). McN-A-343 had a strong potency in the recruitment of Gαq and β-arrestin (EC50 values were 11 nM and 980 nM, respectively), while Pilocarpine showed the lowest potency (EC50 values were 250 μM and 296 μM, respectively). Based on the analysis of the relative intrinsic activity and bias factor (Figure 7), McN-A-343 showed high intrinsic activity and was more likely to recruit Gαq. McN-A-343 is an early example of a relatively selective M1mAChR agonist and is structurally related to commonly used mAChR orthosteric agonists, such as carbachol and oxotremorine-M [28]. Previous studies have reported that McN-A-343 exhibits functional selectivity towards other mAChRs, displaying higher activity in G-protein-mediated signaling pathways compared to CCh [29].

Through molecular docking, we gained insight into the binding mechanism of McN-A-343. It was observed that its binding pocket is higher and closer to the TM extracellular interface compared to endogenous ACh. The 3-chlorophenyl carbamate moiety of McN-A-343 was found to be compatible with Y404 in the orthosteric pocket, leading to the formation of a π–π conjugation and hydrophobic interactions. The position of its docking pocket is similar to that of M2mAChR (M2 muscarinic acetylcholine receptor, M2mAChR) with Iperoxo, as observed in an earlier study [30,31]. In particular, the TM5, TM6, and TM7 helices include key residues that contribute to hydrophobic contact between M1mAChR (active and inactive state) and McN-A-343. The cryo-EM structure of the M1mAChR-Gα11 complex revealed that TM5 extends inward to the β6 strand and α4 helix, while Gα11 residues from the TM7 and C-terminus of M1mAChR extend into the groove of Gα11 that is formed by the Ras domain and Gβ;there is also the outward movement of the cytoplasmic side of TM6. TM5, TM6, and TM7 were also found to play a significant role in Gα11 selectivity [23]. The interaction of the trimethylbutyne of McN-A-343 with M1mAChR causes A196^5.46^, Y381^6.51^, and N382^6.52^ to rotate more towards the cytoplasmic side, thereby inducing an outward expansion of the cytoplasmic side of TM5 and TM6; this also increases the distance between TM5 and TM6 (Figure 8A,B). The TM5 may extend inward to Gαq. The chlorophenyl of McN-A-343 induces Y404^7.39^ and Y408^7.43^ on TM7 to move closer to TM2 and TM3, thus expanding the cytoplasmic side of TM7 and promoting the upward movement of H8. Recently found MOR–Gαi complex structures created by MD provide an explanation for the specific coupling of Gαi proteins that is observed [32]. The carboxy-terminus of the helix is pointed towards TM6 in the structure of the MOR–Gαi1–protein complex, and the only other negative residue, D350, is pointed downwards towards the solvent and away from the TM7/TM2 interface. In addition, the α5 helix has a glycine in the spot closest to TM7, minimizing steric clashes between TM7 and the G-protein as TM7 moves towards TM2 [32]. This study has found that the Gαi-biasing ligand of KOR disrupts the K227^5.39^-E297^6.58^ salt bridge, resulting in an increased distance between TM5 and TM6, and hence a more biased recruitment of Gαi [33]. Therefore, it is hypothesized that the interaction between McN-A-343 and the residues on TM5 and TM7 induces conformational changes that affect the Gαq receptor selectivity.

Iperoxo, which is the orthosteric component of dualistic compounds, is a highly potent muscarinic agonist that contains an affinity-enhancing Δ2-isoxazoline ring system [34]. It was found to be approximately 100 times more potent than ACh, which is consistent with our findings (Figure 2E and Figure 4E), and exhibited a strong preference towards Gαq. Xanomeline, an oral muscarinic cholinergic receptor agonist, also displayed superior intrinsic activity (Figure 7) and preferentially stimulated M1mAChR [35]. As the active state of M1mAChR, the binding pocket is very similar to that of McN-A-343 and can easily adopt an upward extension of the tail in the active conformation, a finding that is in line with the results of recent investigations using M2mAChR [12]. The interaction between Iperoxo and Xanomeline with M1mAChR is primarily mediated by Y404, which is the same for McN-A-343, and this could be the main contributor to their preference for Gαq.

Pilocarpine is a natural alkaloid [36] and is comparatively less effective in recruiting Gαq and β-arrestin2 compared to other agonists (Figure 2 and Figure 4). However, the bias factor (Figure 7) indicated that Pilocarpine increased β-arrestin2 recruitment. Moreover, the analysis of the relative intrinsic activity revealed that β-arrestin was significantly higher (*p* < 0.001) (Table 1), and its signal response to β-arrestin was the highest (Figure 4I), showing a 12% relative increase in Ach. These findings support the hypothesis that Pilocarpine may exhibit a bias towards arrestin, which was also reported by Pronin et al. in 2017 [37]. Additionally, Anja Flöser et al. demonstrated that Pilocarpine showed a relative bias towards β-arrestin3 binding, making it the best recruiter of β-arrestin3 on M3mAChR, but the worst recruiter of GRK2 and Gαq [13]. Recent research has shown that the displacement of TM6 in M2mAChR can accommodate the finger ring of β-arrestin1, which occupies a position similar to the α5 helix of the G protein Ras domain [30]. In M1mAChR, Y381 and W378 are located on TM6, and their conformational changes may induce the displacement of TM6, leading to a bias towards β-arrestin.

Our study revealed that certain agonists can selectively activate Gαq and recruit β-arrestin2 to the receptor, a result that is consistent with previous findings obtained by Suomivuori and Wingler [38,39]. The alternative conformation of AT1R was found to bind β-arrestins but not Gαq, and the positioning of Y292 on TM7 towards TM3 was found to contribute to this conformation. Additionally, we identified two residues, N111 and L112, on TM3, which were tightly coupled to the ligand. Our results indicate that S109 and Y106, which are located at TM3, form hydrogen bonds and hydrophobic bonds with Pilocarpine. Pilocarpine induces a downward (towards intracellular) translation of W378^6.48^ compared to McN-A-343. Pilocarpine contains two rings (an imidazole and furanone ring), which provide a bulkier and more rigid group in the central pocket, impacting the position of W378^6.48^. The interaction between Pilocarpine with Y381^6.51^ and Y408^7.43^ causes the aromatic ring to rotate, which in turn leads to the rotation of TM7, resulting in its signaling bias. The interaction between the β-arrestin biasing ligand and Y326^7.43^ in μOR causes a rotation of TM7, resulting in a more biased pocket [40]. The residues interacting with Pilocarpine, such as Y106, S109, Y381, and W378, are different from those of the Gαq-biased agonists, such as McN-A-343, which may trigger different signaling biases by affecting the TM of M1mAChR. In M1mAChR, the G protein-biased ligand McN-A-343 positions the aromatic ring between TM7 and TM6, whereas the arrestin-biased ligand Pilocarpine typically favors TM3. Studies have shown that placing bulky ligands in the TM2-TM3 subpocket favors β-arrestin protein signaling, while leaving this subpocket unoccupied favors G protein signaling [41]. Recent studies by MD have found that the β-arrestin-biased ligand of KOR interacts with Q115^2.60^ and W287^6.48^ to jointly control the rotation of TM7, inducing W287^6.48^ to rotate towards the inner side of the cell [33], which is consistent with the changes induced by Pilocarpine on W378^6.48^ in our study.

The binding of G proteins and β-arrestin can be differentially affected by various receptors and ligands, which opens up possibilities for the development of drugs that can specifically direct signaling in one direction or another. Our findings highlight the importance of considering M1mAChR ligand induction bias in distinguishing between Gαq recruitment bias and β-arrestin recruitment bias. This strategy could be applied to all GPCRs, G proteins, and β-arrestin, providing new insights into biased pathways at the level of effector protein recruitment and activation.

## 4. Materials and Methods

### 4.1. Cell Culture

HEK-293T cells were obtained from ATCC, while CHO-M1 cells were purchased from Genscript. The HEK-293T cells were cultured and transfected with plasmids in DMEM (BasalMedia, Shanghai, China) medium containing 10% FBS (LONSERA, Canelones, Uruguay), 100 U/mL of penicillin, and 100 µg/mL of streptomycin (Gibco™, Thermo Scientific, Waltham, MA, USA) in a humidified atmosphere at 37 °C with 5% CO_2_. After transfection, the cells were plated on DMEM supplemented with 10% FBS for BRET assays. The CHO-M1 cells were maintained and transfected in F12 medium (Gibco™, Thermo Scientific, Waltham, MA, USA) containing 10% FBS, 100 U/mL of penicillin, 100 µg/mL of streptomycin, and 0.1% Zeocin™ (Thermo Scientific, Waltham, MA, USA) in a humidified atmosphere at 37 °C with 5% CO_2_. After transfection, the cells were plated on F12 medium supplemented with 10% FBS for BRET assays. 

### 4.2. cDNA Constructs

The cDNA for M1mAChR-Rluc8, with Rluc8 fused to the C-terminus of M1mAChR [42], was obtained, while the cDNA for Gαq Gβ2 was sourced from the PubMed cDNA Resource Center and pcDNA3.1+ was obtained from Invitrogen. Gγ2-mVenus, with mVenus fused to the N-terminus of Gγ2 [43], and β-arrestin2-mVenus, with mVenus fused to the C-terminus of β-arrestin2 [44], were also included. The plasmid constructs were purchased from Sangon Biotech (Shanghai, China).

### 4.3. BRET Assays for Gαq and β-Arrestin Coupling

The method was as follows: Maintain HEK-293T cells in a 25 cm^2^ culture flask containing 5 mL of culture medium at 37 °C with 5% CO_2_. After 24 h, seed trypsinize cells at a density of 10^4^ cells/100 μL per well on a black flat-bottomed 96-well plate (Corning Inc., Corning, NY, USA, Cat. No. 3606). Incubate the cells for another 24 h at 37 °C with 5% CO_2_ until the cell density reaches 70%~80%. To transfect the cells, use expression constructs (total 0.1 μg/well) with a 1:1:1:1 DNA ratio of M1-RLuc8/Gαq/Gβ2/Gγ2-mVenus, or a 1:2 DNA ratio of M1-RLuc8/β-arrestin2-mVenus, and LipofectamineTM 3000 (0.15 µL/well) reagents (Thermo Scientific, Waltham, MA, USA, Cat. No. R25001). Incubate the cells for another 24 h at 37 °C with 5% CO_2_. Aspirate the medium and wash the cells three times with 100 µL Tyrode solution (Tyrode solution containing 150 mM of NaCl, 4 mM of KCl, 2 mM of MgCl_2_, 2 mM of CaCl_2_, 20 mM of HEPES, and 10 mM of glucose, with a pH of 7.4). Add Tyrode solution and a given type of agonist solution (ACh, CCh, Iperoxo, Xanomeline, Pilocarpine, and McN-A-343 purchased from MedChemExpress, Monmouth-Junctio, NJ, USA)to the cells. Then, add 10 µL of freshly prepared luciferase substrate (here, Coelenterazine h was added at a final concentration of 5 μM, Hangzi Biotechnology, Shanghai, China) and mix the solution. To determine the BRET ratio, activate the microplate reader (Pherastar FSX, BMG LABTECH, Offenburg, Germany), adjust the filters to BRET, and measure the dual emissions at 475 nm and 530 nm wavelengths, as described in the previous study [45].

### 4.4. Fluo-4-AM

The influx of intracellular calcium was determined using Fluo4-AM (DOJINDO, Tonomachi, Japan, Cat. No.F311). CHO-M1 cells were seeded on 96-well plates at a density of 40,000 cells/well. The cells were treated with 5 µM of Fluo4-AM in 5 mM of CaCl_2_ buffer and incubated for 30 min at 37 °C in complete darkness. After incubation, the cells were washed twice with HBSS (BasalMedia, Shanghai, China), and the fluorescence was measured by exciting Fluo4 at 488 nm and detecting the emitted light at 520 nm.

### 4.5. β-Arrestin Recruitment

CHO-M1 cells were seeded on a white opaque 96-well microplate (PerkinElmer-cisbio, Waltham, MA, USA, Cat. No. 6005680) at a density of 40,000 cells/well and incubated at 37 °C with 5% CO_2_. We used the β-arrestin2 recruitment KIT test (PerkinElmer, Waltham, MA, USA, Cat. No. 62BDBAR2PEB). Following 24 h of incubation, the cell culture medium was removed, and Stimulation Buffer 4 was added to the negative control group and the blank group, while a diluted agonist was added to the agonist group, at a volume of 100 µL for each group. The plates were then sealed and allowed to incubate at room temperature for 15 min. Then, we removed the Stimulation Buffer 4 from the wells and added 30 µL of Stabilization Buffer 1, sealed the plate, and incubated it at room temperature for 15 min. Afterward, Stabilization Buffer 1 was removed, and the wells were washed three times with 100 µL of Wash Buffer 1. Following that, 100 µL of pre-mixed d2 and Eu cryptate antibodies were added to the wells, and the plates were sealed and incubated overnight at room temperature. Finally, the plates were read using an HTRF-compatible reader.

### 4.6. Molecular Docking

The 2D structure of the ligands (ACh, CCh, Iperoxo, Xanomeline, Pilocarpine, and McN-A-343) were drawn using ChemDraw (CambridgeSoft, Cambridge, MA, USA)and subsequently imported into ChemDraw 3D for energy minimization using the MM2 module to obtain the lowest energy conformation and saved as a mol2 file. The protein structures were downloaded from the PDB database (PDB ID: 5CXV for inactive state and 6OIJ for active state) and subsequently visualized separately using PyMol (Schrodinger, New York, NY, USA), and then the ligands and receptors were saved as pdbqt files using Mgtools 1.5.6 after processing via dehydration, hydrogen addition, charge calculation, merging with non-polar hydrogen, etc. The ligands and receptors were docked using Autodock vina 1.1.2 (TSRI, La Jolla, CA, USA)with the docking parameters shown below: num-modes = 9, energy-range = 3, and exhaustiveness = 8. The highest-scoring conformation was visualized with PYMOL.

### 4.7. Data Analysis

All the concentration–response curves were fitted to a three-parameter logistic equation in Prism 8.0 (GraphPad Software, San Diego, CA, USA). The BRET concentration–response curves were analyzed by either conducting normalization to a reference agonist for each experiment or by analyzing them as raw net BRET (fit Emax-fit baseline). The EC50 and Emax values were estimated by fitting all biological replicates simultaneously. The BRET ratio was calculated using the following formula: BRET ratio = (emission of mVenus at 535 nm with a 30 nm band path width)/(emission of Rluc8 at 475 nm with a 30 nm band path width)–(emission of Rluc8 at 535 nm)/(emission of Rluc8 at 475 nm). For a comparison of the agonist effects of different signaling pathways, the relative intrinsic activity (RAi) was calculated according to Griffin, Figueroa, Liller, and Ehlert [29]: RAi = (τ_ACh_ × KA_a_)/(τ_a_ × KA_ACh_), where τ_a_ and KA_a_ are the half-effective concentration and apparent maximal response to the tested compound, respectively. To estimate the putative signaling bias between the Gαq and β-arrestin pathways, the ΔΔlog(τ/KA) method was used, based on the following equation: bias = 10^ΔΔlog(τ/KA) Gαq−β-arrestin^ [46], which is derived from the RAi equation [29]. Here, ΔΔlog (τ/KA) _Gαq−β-arrestin_ = Δlog(τ/KA) _Gαq_ − Δlog(τ/KA) _β-arrestin_. For all statistically analyzed studies, the experiments were performed at least three times independently. The experiments were carried out in a randomized manner. The results are presented as the mean ± SEM. Statistical analysis was performed with a one-way analysis of variance (ANOVA). A value of *p* < 0.05 was considered statistically significant.

## Figures and Tables

**Table 1:** The relative intrinsic activity of agonists towards signaling pathways.

Ligand	Gαq (BRET) (Δlog (τ/KA))	β-Arrestin (BRET) (Δlog (τ/KA))	Gαq (Fluo4-AM) (Δlog (τ/KA))	β-Arrestin (FRET) (Δlog (τ/KA))
McN-A-343	1.85 ± 0.010	0.34 ± 0.040	1.20 ± 0.005	−0.67 ± 0.020
Iperoxo	1.56 ± 0.090	1.27 ± 0.020	0.98 ± 0.003	−0.03 ± 0.040
Xanomeline	1.58 ± 0.050	0.94 ± 0.030	0.32 ± 0.002	−0.29 ± 0.020
CCh	0.26 ± 0.020	0.26 ± 0.040	−1.09 ± 0.030	−1.12 ± 0.040
Pilocarpine	−3.20 ± 0.001	−2.70 ± 0.002	−2.61 ± 0.002	−2.08 ± 0.030

## Data Availability

Not applicable.

## Abbreviations

GPCRs	G-protein-coupled receptors
M1mAChR	M1 muscarinic acetylcholine receptor
BRET	Bioluminescence resonance energy transfer
FRET	Fluorescence resonance energy transfer
ACh	Acetylcholine
CCh	Carbachol
RAi	Relative intrinsic activity
